# An Anthropometric Risk Index Based on Combining Height, Weight, Waist, and Hip Measurements

**DOI:** 10.1155/2016/8094275

**Published:** 2016-10-18

**Authors:** Nir Y. Krakauer, Jesse C. Krakauer

**Affiliations:** ^1^Department of Civil Engineering, The City College of New York, New York, NY, USA; ^2^Metro Detroit Diabetes and Endocrinology, Southfield, MI, USA

## Abstract

Body mass index (BMI) can be considered an application of a power law model to express body weight independently of height. Based on the same power law principle, we previously introduced a body shape index (ABSI) to be independent of BMI and height. Here, we develop a new hip index (HI) whose normalized value is independent of height, BMI, and ABSI. Similar to BMI, HI demonstrates a U-shaped relationship to mortality in the Third National Health and Nutrition Examination Survey (NHANES III) population. We further develop a new anthropometric risk index (ARI) by adding log hazard ratios from separate nonlinear regressions of the four indicators, height, BMI, ABSI, and HI, against NHANES III mortality hazard. ARI far outperforms any of the individual indicators as a linear mortality predictor in NHANES III. The superior performance of ARI also holds for predicting mortality hazard in the independent Atherosclerosis Risk in Communities (ARIC) cohort. Thus, HI, along with BMI and ABSI, can capture the risk profile associated with body size and shape. These can be combined in a risk indicator that utilizes complementary information from height, weight, and waist and hip circumference. The combined ARI is promising for further research and clinical applications.

## 1. Introduction

Body mass index (BMI) (weight [*W*] relative to height [*H*] as *W*/*H* ^2^) [1] has been robustly established to be independent of height in numerous and diverse population studies and is currently used in the definition of overweight and obesity. Waist circumference (WC) has been used to indicate the presence of abdominal obesity, with WC above threshold forming one criterion for diagnosis of metabolic syndrome [2, 3]. However, the high correlation (0.8–0.9) found between BMI and WC or WC-derived measures such as WC/*H* ratio [4–6] and body roundness index [7, 8] limit the utility of these measures beyond BMI.

BMI traces back to the pioneering 1800s statistician Quetelet, who postulated a power-law relationship between height and weight [9]. BMI can be considered a special case of the concept of power-law scaling of body dimensions (allometry), developed in biology during the early 1900s [10]. Building on these ideas, we previously applied regression based on power-law scaling to derive an index (a body shape index [ABSI]) that expresses waist circumference (WC) relative to height and weight and was therefore statistically independent of BMI [11]. Odds ratios for mortality in several longitudinal studies showed a U-shaped distribution across BMI and a positive linear association with ABSI [12–14].

Hip circumference (HC) and derived measures such as waist to hip ratio (WC/HC, WHR) have also been studied extensively as risk factors [15–17]. However, HC and WHR are typically highly correlated to BMI or WC, and several studies have failed to show added value of HC-based indicators compared to those only based on *H*, *W*, and WC [18–20].

In general, comparisons of various indices based on *H*, *W*, WC, and HC have shown that different individual indices may perform approximately equally well as predictors of mortality and conditions such as heart disease [14, 20–25]. While the joint use of multiple indices could improve risk prediction, high correlations between different measures are one reason that there is as yet no clear methodology to obtain combinations of indicators that outperform the ones currently used [26–28].

Here, we propose a new approach to transform *H*, *W*, WC, and HC to an anthropometric risk index (ARI). The first step in this approach was to reexpress *H*, *W*, WC, and HC data as derived indicators that are almost uncorrelated with each other. The proposed set of independent indicators includes *H*, BMI, ABSI, and a newly developed hip index (HI).

We use nonlinear (penalized spline) regression with data from the Third National Health and Nutrition Examination Survey (NHANES III), a United States (USA) general population sample with some 20 years of follow-up for mortality, to estimate a functional relationship between each indicator and mortality hazard within the Cox proportional hazard framework. ARI is formed by summing the estimated logarithms of hazard ratios due to each of the independent indicators and constitutes a linear predictor for logarithm of mortality hazard. We demonstrate that ARI is transferable beyond the cohort in which it is developed by applying it to mortality outcomes of a different USA cohort study, Atherosclerosis Risk in Communities (ARIC). The ARI approach developed here could be applied to produce risk indices for various conditions that make maximum use of readily obtained body measurements for research and clinical decision-making.

## 2. Methods

### 2.1. Ethics Statement

We analyzed data from the NHANES III and ARIC studies. The NHANES III and ARIC protocols were approved by the NHANES Institutional Review Board and the University of North Carolina at Chapel Hill Office of Human Research Ethics, respectively, and all participants gave written informed consent [29, 30]. The present analysis of the already-anonymized public-use data from these studies was approved as exempt from review by the University Integrated Institutional Review Board of the City University of New York.

### 2.2. Data

NHANES III sampled the civilian noninstitutionalized USA population using a cluster approach, with some groups of public health interest (children, the elderly, and black and Mexican-American people) deliberately oversampled [31]. The data collected were used to study the prevalence of health behaviors and risk factors, including anthropometric parameters [27, 32–35]. Subjects were interviewed and examined during 1988–1994. Linked mortality outcomes for adult subjects were available from the National Center for Health Statistics with follow-up through 2011 (17–23 years of follow-up). These mortality outcomes were derived from probability matching with the National Death Index, with those not matched to a death record assumed to have stayed alive through the end of the period. For NHANES III, height was measured with a stadiometer, weight with a digital scale, WC with a steel tape about the high point of the iliac crest at minimal respiration, and HC at the maximum extension of the buttocks [36]. We analyzed NHANES III public-use data for all nonpregnant adults (age 18 and over) with *H*, *W*, WC, and HC measurements and mortality follow-up.

The ARIC cohort component sampled 4,000 adults (age 45–64) in each of the 4 USA communities during 1987–1989 to study correlates of heart disease risk [29, 37–39]. ARIC participants were visited several times in subsequent decades for follow-up examinations [40]. We obtained ARIC public-use repository data v2015a from the National Institutes of Health Biologic Specimen and Data Repository Information Coordinating Center (BIOLINCC), containing follow-up for mortality outcomes through 2010 (21–23 years of follow-up). We analyzed the ARIC data for all adults with initial *H*, *W*, WC, and HC measurements and mortality follow-up.

Except where otherwise specified, we used the provided sample weights [31] in all analyses of the NHANES III data so that our results would be better estimates for anthropometric normals and correlations with mortality hazard in the general USA population. The ARIC analyses weight all participants equally.

### 2.3. Analysis

*H*, BMI, and ABSI have been found to be mutually almost uncorrelated (correlation coefficient magnitudes |*r*| < 0.1) in several cohorts [13]. To obtain a fourth index uncorrelated with these three, we followed a procedure similar to that used to construct ABSI [11], seeking a power-law relationship between HC (cm) and *H* (cm) and *W* (kg), adjusted for sex, in NHANES III nonpregnant adults by linear regression of the natural logarithms. The least-square regression line was

(1) log⁡HC=2.658−0.310log⁡H+0.482log⁡W+0.083f,

where the indicator *f* is set at 1 for females and 0 for males (*R* ^2^ = 0.887). Based on this relationship, we defined a normalized HC, or HI, as

(2) $$\begin{array}{c} HI\equiv HC{\left(\;\frac{H}{\langle H\rangle }\;\right)}^{0.310}{\left(\;\frac{W}{\langle W\rangle }\;\right)}^{-0.482}, \end{array}$$

where 〈*H*〉 = 166 cm and 〈*W*〉 = 73 kg were average values. HI can be understood as the HC of a given person normalized to a standard height and weight (ABSI could also be expressed in an analogous normalized form and denote WC normalized to a standard height and weight).

Cox proportional hazard modeling [41] was used to assess the impact of the anthropometric indices *H*, BMI, ABSI, and HI on death rate (mortality hazard) over the follow-up periods [13]. In addition to body measurements, age (used as the timescale in the Cox model), sex (male/female), and race (black/nonblack) were also retained for modeling mortality hazard. Body measurements were normalized to age- and sex-specific *z* scores [13] based on NHANES III means and standard deviations before being entered into the model. These *z* scores for *H*, BMI, ABSI, and HI were found, in both NHANES III and ARIC, to indeed be mutually almost uncorrelated, whereas WC and HC had high correlations (about 0.9) and WHR had moderate correlation (about 0.4–0.5) with BMI (Tables 1 and 2).

Both linear and nonlinear associations with mortality hazard were modeled for each index for both NHANES III and ARIC and compared to a baseline model with only sex and race as predictors. In the linear proportional hazard models, death rate increases or decreases by a constant factor per standard deviation change in the index (unit change in *z* score). Nonlinear associations were estimated using a penalized spline basis, with the corrected Akaike information criterion (AIC) used to choose the amount of smoothing [42–44].

The main measure of relative model performance was AIC difference score, Δ_*i*_. For the best-performing model (with lowest AIC), Δ_*i*_ = 0, while other models have positive Δ_*i*_ [45]. Δ_*i*_ > 6 indicated models that perform significantly worse than the best-performing model (at the 95% confidence level) as mortality predictors for the sampled population [13]. We also calculated coefficients *R* ^2^, denoting the proportion of variation in mortality explained by the predictors of each model, so that higher *R* ^2^ suggests a model with more explanatory power [46]. Another measure of model performance checked was concordance (*C*), defined as the fraction of pairs of individuals in the sample for which the one modeled to be at greater risk actually died sooner [41]. Concordance ranges from 0 to 1, with 0.5 being the expected value for models with no skill and higher values denoting models that are more skillful at explaining variation in survival. Regressions were carried out in the R environment [47], using the survival [48] package for the model fitting and calculation of AIC and *C* scores and the survMisc [49] package for calculating model *R* ^2^.

Nonlinear modeling for mortality hazard associated with each anthropometric index yielded functions for the natural logarithm of the estimated hazard for different values of the *z* score of that particular index. ARI was taken to be the sum of these function values for each individual's combination of anthropometric index *z* scores, denoting the natural logarithm of the combined estimated hazard from the four independent indices* H*, BMI, ABSI, and HI. Assuming that these four hazards are independent, ARI then is the natural logarithm of the mortality hazard based on all four measurements* H*,* W*, WC, and HC, with positive values denoting above-average combined risk and negative values denoting lower risk. ARI calculated based only on NHANES normals and outcome data was applied to the ARIC population to test whether it is transferable beyond the original cohort used to obtain the population normals and hazard estimates.

## 3. Results

While the ARIC cohort was on average older than NHANES III or the national adult population, the groups had fairly similar body measurements on initial examination, with most individuals in the overweight or obese BMI ranges (Table 3). Associations with mortality hazard of* H*, BMI, ABSI, and HI *z* scores were also broadly similar for the NHANES III and ARIC cohorts, though there were significant differences in detail. Out of* H*, BMI, ABSI, and HI, the best linear predictor for log mortality was ABSI for both datasets. BMI was a weaker but also statistically significant linear predictor for both cohorts, while HI was only statistically significant as a linear predictor in ARIC, and* H* was only marginally significant for both cohorts (Tables 4 and 5).

**Figure 1:**
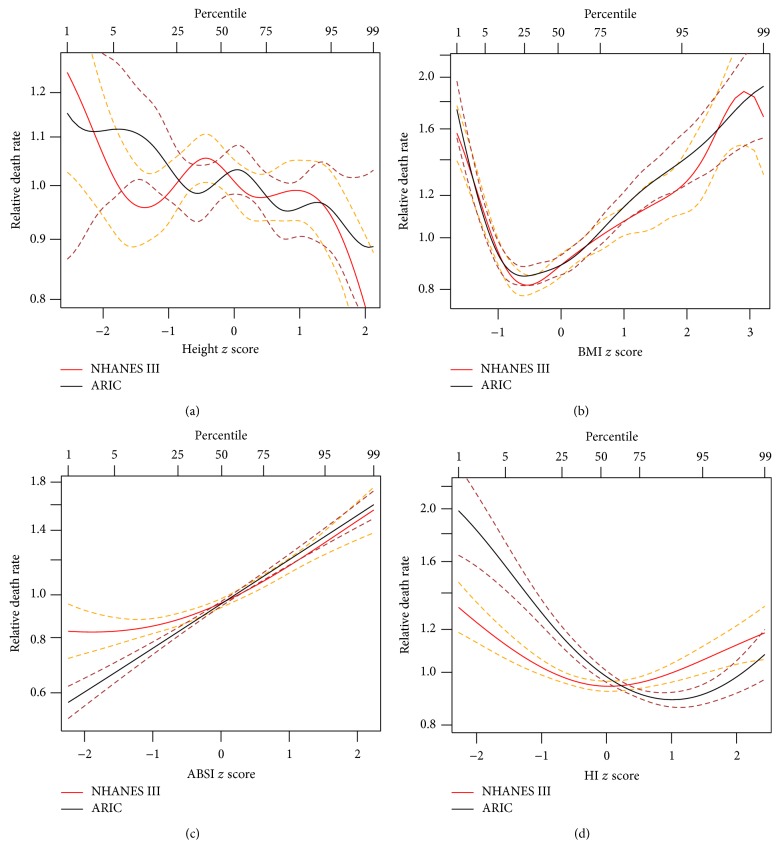
Estimated mortality hazard ratios in NHANES III and ARIC as nonlinear (penalized spline) functions of normalized (a) height, (b) BMI, (c) ABSI, and (d) HI. Dashed lines indicate 95% confidence intervals. Percentiles and *z* scores are based on the NHANES III cohort.

In nonlinear (penalized spline) regression models, BMI and HI showed asymmetric U-shaped associations with mortality in both cohorts, while associations with* H* and ABSI were basically monotonic. The optimum BMI (lowest mortality hazard) was around half a standard deviation under the population median (in the World Health Organization “normal” category) for both NHANES III and ARIC. However, the optimum HI was higher for ARIC than for NHANES III (Figure 1). As measured by Δ_*i*_ as well as *R* ^2^ and *C*, ABSI and BMI had the strongest nonlinear associations with mortality in both cohorts (Tables 4 and 5).

In NHANES III, ARI was a significantly better linear predictor than any of the (linear or nonlinear) models based on individual indicators (*H*, BMI, ABSI, and HI) (Table 4). ARI derived from NHANES III data predicted mortality hazard for the ARIC cohort better than any model based on one of the individual indicators (Table 5), despite the difference in detail in the anthropometry associations with mortality hazard of the two cohorts.

## 4. Discussion

The difference in optimum BMI found between the NHANES III and ARIC cohorts, on the one hand, and the more recently enrolled NHANES 1999–2004 cohort, on the other hand [11], is consistent with variations in optimum BMI seen between different population studies [25, 50–58]. The U-shaped association of mortality hazard with relative hip circumference (HI *z* score) has not been reported before, to our knowledge. The strong performance of ABSI, compared to other anthropometric indices, as a near-linear predictor of log mortality hazard is consistent with analyses of other cohorts [12–14, 25]. Our results suggest that these associations of mortality risk with different measures of body size and shape can be combined in a single robust linear predictor, ARI.

Previously, anthropometric indices have not included all four of the measurements* H*,* W*, WC, and HC, although each of these measurements has been separately found to be a significant determinant of health risk in various studies. The ARI approach can combine the risk associated with these four measurements and potentially also others such as thigh circumference [59], X-ray body composition measures [60, 61], or body dimensions from laser scanning [62, 63].

Although our ARI is a substantially better predictor of mortality risk than any of the individual anthropometric indices tested, its absolute predictive value is modest for the cohorts and follow-up periods tested: the measure of explained variation *R* ^2^ increases by about 0.04, while concordance increases by about 0.03–0.06, relative to a background predictive model with no anthropometric data (Tables 4 and 5). In fact, self-reported health and smoking history have been found to be the best single predictors of 5-year mortality [64], probably outperforming any anthropometrics. The ARI approach could provide a way to incorporate such indicators as well as laboratory measurements and anthropometric indices into reliable combined risk estimates, expressing these in terms of statistically independent components whose attributable risks can then be summed.

Commonly, WC but not HC is measured. In such cases, ARI could be modified to sum only risk due to the indicators* H*, BMI, and ABSI. Note that, in both the NHANES III and ARIC cohorts, BMI and ABSI are better nonlinear indicators of mortality hazard compared to HI and* H* (Tables 4 and 5), suggesting that this truncated ARI would retain most of the predictive power of the fuller version used here.

One potential drawback of ARI as calculated from cohorts such as NHANES III is that it is not a simple function of* H*,* W*, WC, and HC, so that determining its value for a particular individual from these measurements would require either lookup tables or a computer program. This complexity could be overcome as an obstacle to clinical use via online calculators (analogous to the calculator developed for estimating combined risk from BMI and ABSI [11, 65], currently available online at https://nirkrakauer.net/sw/absi-calculator.html) or apps for desktop and mobile device use. We have developed a prototype online calculator for computing NHANES III ARI from* H*,* W*, WC, and HC values, available at https://nirkrakauer.net/sw/ari-calculator.html. The NHANES III population means and standard deviations and risk curves needed to carry out this ARI computation are also available in spreadsheet form as a supplement to this article (in Supplementary Material available online at http://dx.doi.org/10.1155/2016/8094275).

The work presented here has several limitations that could be addressed in future studies. The quality of the mortality follow-up information from NHANES III and ARIC has not been, to our knowledge, rigorously verified, raising the possibility of some bias in the estimated risks, although the consistency across the two cohorts of the associations of mortality with initially measured anthropometric variables is reassuring. The data we use here is only from USA. It is likely that the NHANES III derived ARI should be modified for application to non-USA populations [66]; this could be done by calibration to other large cohort studies with mortality follow-up. To assess the usefulness of combined indices for specific clinically used classification schemes and decisions, performance measures such as net reclassification improvement (NRI) and integrated discrimination improvement (IDI) that are based on particular concepts of value to clinical decision-making [67, 68] could be calculated.

The approach used to compute ARI here for hazard of all-cause mortality could also be extended to derive risk indices customized for specific causes of death and morbidity outcomes such as heart disease, stroke, or diabetes, which could facilitate individualized cost-benefit consideration in deciding what medical interventions to undertake [69–71]. One recent study found that anthropometric indices (ABSI and WC/*H* ratio) were correlated with Framingham and SCORE 10-year cardiovascular risk estimates in a nationally representative Turkish sample [72], while another study found anthropometry-based indices (specifically ABSI) to predict cardiovascular disease in middle-aged and elderly Dutch adults as well as a risk model that included laboratory measurements [73], highlighting the potential for utilization of a combination of several readily obtained body measurements for cardiometabolic risk assessment.

## 5. Conclusions

We derived and tested a combined anthropometric risk index that takes into account multiple body measurements to arrive at a risk score that outperforms the individual indices previously used. The allometry-inspired methodology used to arrive at the components of this index can potentially be applied to define mutually independent indices from a broad range of biometric and other variables and has the potential to help elucidate the findings from association and observational studies.

## Supplementary Material

Spreadsheet containing tables for (a) the age and sex specific population means and standard deviations of height, BMI, ABSI, HI (used to transform their raw values to z scores); (b) conversion from height, BMI, ABSI, HI z scores to population percentiles; (c) conversion from percentile to relative mortality hazard. All these tabulated values are based on the NHANES III data analyzed in the paper.

## Figures and Tables

**Table 1:** Correlations of body measures in NHANES III.

	Height	Weight	BMI	WC	HC	ABSI	WHR	HI
Height	1	0.486	−0.010	0.213	0.079	0.074	0.252	−0.528
Weight	0.360	1	0.863	0.872	0.821	0.115	0.413	−0.251
BMI	−0.011	0.919	1	0.876	0.905	0.083	0.413	0.021
WC	0.152	0.904	0.905	1	0.791	0.506	0.736	−0.154
HC	0.247	0.933	0.903	0.867	1	0.040	0.173	0.319
ABSI	0.059	0.028	0.007	0.385	0.030	1	0.777	−0.132
WHR	−0.056	0.415	0.468	0.699	0.260	0.717	1	−0.599
HI	0.016	0.034	0.031	0.028	0.356	0.030	−0.459	1

Correlation coefficients for body measures among NHANES III nonpregnant adults. The upper-right triangle of the table shows correlations of the raw values, while the lower-left triangle shows correlations of the *z* scores relative to age- and sex-specific means.

**Table 2:** Correlations of body measures in ARIC.

	Height	Weight	BMI	WC	HC	ABSI	WHR	HI
Height	1	0.471	−0.053	0.165	0.009	0.031	0.279	−0.560
Weight	0.322	1	0.851	0.862	0.793	0.048	0.525	−0.308
BMI	−0.067	0.747	1	0.882	0.900	0.038	0.429	−0.015
WC	0.089	0.706	0.894	1	0.821	0.455	0.724	−0.113
HC	0.191	0.790	0.893	0.855	1	0.070	0.205	0.307
ABSI	0.059	−0.009	0.041	0.450	0.082	1	0.691	0.038
WHR	−0.057	0.287	0.493	0.733	0.284	0.724	1	−0.571
HI	0.010	0.009	−0.049	−0.009	0.317	0.088	−0.442	1

The same as Table 1, but for ARIC.

**Table 3:** NHANES III and ARIC means.

	NHANES III	NHANES III (weighted)	ARIC
Number	16034		14917
Deaths	4897		4829
% female	52	51	51
% black	28	11	24
Age (y)	43 (30–63)	41 (30–57)	54 (49–59)
Height (cm)	166 (159–174)	168 (161–176)	168 (161–176)
Weight (kg)	73 (63–85)	73 (62–85)	78 (67–89)
BMI (kg m^−2^)	26 (23–30)	25 (22–29)	27 (24–30)
WC (cm)	92 (82–102)	90 (80–101)	96 (88–105)
HC (cm)	99 (93–106)	99 (93–106)	103 (98–109)
WHR	0.92 (0.85–0.98)	0.91 (0.84–0.97)	0.94 (0.88–0.98)
ABSI (m^11/6^ kg^−2/3^)	0.0803 (0.0764–0.0841)	0.0798 (0.0761–0.0834)	0.0823 (0.0792–0.0852)
HI (cm)	100 (96–105)	100 (96–105)	102 (98–106)

Comparison of demography and body measurements in the NHANES III and ARIC cohorts. For age and for body measurements, medians and interquartile ranges are given. For NHANES III, frequencies and quantiles were also calculated with the sample weights given to better represent the national population.

**Table 4:** Mortality hazard association with body measures in NHANES III.

Predictor	Hazard ratio per SD increase	Δ_*i*_	*R* ^2^	*C*
ARI (linear)	1.46 (1.41–1.52)	0	0.065	0.615
BMI (nonlinear)		187.2	0.046	0.591
ABSI (nonlinear)		247.3	0.036	0.585
ABSI (linear)	1.16 (1.12–1.20)	256.4	0.034	0.584
HI (nonlinear)		314.6	0.028	0.567
BMI (linear)	1.07 (1.04–1.10)	326.4	0.025	0.570
*H* (nonlinear)		327.5	0.027	0.569
*H* (linear)	0.96 (0.93–0.99)	336.4	0.024	0.562
None		342.8	0.023	0.555
HI (linear)	0.99 (0.96–1.02)	343.9	0.023	0.558

Results of Cox proportional hazard modeling for mortality risk in NHANES III with *H*, BMI, ABSI, HI, or ARI *z* scores taken as linear or nonlinear predictors. All models also included as predictors sex and race. Ranges in parentheses are 95% confidence intervals for the hazard ratio. Models are arranged in decreasing order of skill (increasing Δ_*i*_).

SD: standard deviation; Δ_*i*_: Akaike information criterion score difference relative to the best-performing model shown (see Methods for details); *R* ^2^: measure of explained variation; *C*: concordance.

**Table 5:** Mortality hazard association with body measures in ARIC.

Predictor	Hazard ratio per SD increase	Δ_*i*_	*R* ^2^	*C*
ARI (linear)	1.43 (1.38–1.49)	0	0.103	0.622
ABSI (linear)	1.26 (1.22–1.30)	86.0	0.093	0.616
ABSI (nonlinear)		86.4	0.093	0.616
BMI (nonlinear)		121.9	0.090	0.613
HI (nonlinear)		221.5	0.078	0.606
BMI (linear)	1.11 (1.08–1.15)	277.3	0.070	0.601
HI (linear)	0.92 (0.89–0.95)	294.4	0.067	0.602
*H* (linear)	0.96 (0.93–0.99)	312.0	0.065	0.596
None		317.4	0.064	0.593
*H* (nonlinear)		318.8	0.066	0.597

The same as Table 4, but for the ARIC cohort.
